# Near-infrared photothermal/photodynamic therapy with indocyanine green induces apoptosis of hepatocellular carcinoma cells through oxidative stress

**DOI:** 10.1038/s41598-017-14401-0

**Published:** 2017-10-24

**Authors:** Chikara Shirata, Junichi Kaneko, Yoshinori Inagaki, Takashi Kokudo, Masumitsu Sato, Sho Kiritani, Nobuhisa Akamatsu, Junichi Arita, Yoshihiro Sakamoto, Kiyoshi Hasegawa, Norihiro Kokudo

**Affiliations:** Hepato-Biliary-Pancreatic Surgery Division, and Artificial Organ and Transplantation Division, Department of Surgery, Graduate School of Medicine, The University of Tokyo, Tokyo, Japan

## Abstract

Indocyanine green (ICG) is a photothermal agent, photosensitizer, and fluorescence imaging probe which shows specific accumulation in hepatocellular carcinoma (HCC) cells. We recently developed a photodynamic therapy (PDT) using ICG and near-infrared (NIR) laser as a new anti-cancer treatment for HCC. However, the molecular mechanism underlying this effect needs to be elucidated. HuH-7 cells, a well-differentiated human HCC cell line, were transplanted subcutaneously into BALB/c-nu/nu mice for *in vivo* experiment. ICG was administered 24 h before NIR irradiation. The irradiation was performed at three tumor locations by 823-nm NIR laser on days 1 and 7. The temperature of HuH-7 xenografts increased to 48.5 °C 3 minutes after ICG-NIR irradiation start. Reactive oxygen species (ROS) production was detected after ICG-NIR irradiation both *in vitro* and *in vivo*. There was certain anti-tumor effect and ROS production even under cooling conditions. Repeated NIR irradiation increased the cell toxicity of ICG-NIR therapy; the mean tumor volume on day 9 was significantly smaller after ICG-NIR irradiation compared to tumor without irradiation (87 mm^3^ vs. 1332 mm^3^; p = 0.01) in HCC mice xenografts model. ICG-NIR therapy induced apoptosis in HCC cells via a photothermal effect and oxidative stress. Repeated ICG-NIR irradiation enhanced the anti-tumor effect.

## Introduction

Indocyanine green (ICG) has been reported as a photothermal agent, photosensitizer, and fluorescence imaging probe. Near-infrared (NIR) laser-induced photothermal therapy (PTT) uses a photothermal agent to convert optical energy into thermal energy and has the potential as an effective local, minimally invasive anti-tumor treatment^1–3^. ICG has been reported to be an effective NIR light absorber for laser-mediated PTT^4,5^.

Photodynamic therapy (PDT) is a noninvasive treatment that combines a photosensitizer and an activating light source. It involves the administration of a photosensitizer, which leads to selective uptake and retention in tumor cells. After activation by exposure to light of an appropriate wavelength, the photosensitizer releases reactive oxygen species (ROS) and singlet oxygen, which show anti-tumor effects^6,7^. Combined treatment with the photosensitizer indocyanine green (ICG) and near-infrared (NIR) light irradiation was initially used for treating skin lesions^8^.

ICG is also approved by the US Food and Drug Administration (USFDA) as a NIR clinical imaging agent^9–11^. We recently reported that ICG accumulates in hepatocellular carcinoma (HCC) cells, and developed a navigation system for identifying HCC lesions during surgery^12^. Major advantages of ICG are its safety and feasibility, with incidences of adverse reactions of less than 0.01%^13^.

HCC is the most common primary malignancy of the liver and the third leading cause of cancer deaths. Surgical resection, liver transplantation, and ablation therapy are the only curative treatments for HCC. However, liver function in patients with HCC is often impaired due to underlying liver diseases, such as viral hepatitis or alcoholic hepatitis, and curative treatment for patients with multiple tumors is difficult in many cases^14,15^.

The expression of organic anion-transporting polypeptide, which is the uptake transporter for ICG, is preserved in HCC tissues, whereas biliary excretion is impaired because of morphological changes associated with cancer progression, leading to accumulation of ICG after preoperative intravenous administration^13,16,17^.

We previously reported that ICG-NIR PDT suppressed tumor growth in a xenograft model and may be an effective adjuvant therapy for HCC, i.e., for disseminated lesions^18^. However, the detailed mechanisms mediating the inhibition of tumor growth remain unclear. Here we investigated the mechanisms underlying the anti-tumor effect of ICG-NIR therapy.

## Methods

### Animals and cell lines

This study protocol was approved by the University of Tokyo Animal Ethics Committee (I-P 10–118) in accordance with Japanese guidelines and regulations for scientific and ethical animal experimentation. Male BALB/c nude mice were purchased from Charles River Laboratories Japan, Inc. (Yokohama, Japan), and used at 5 to 6 weeks of age (n = 30, mean weight 18 g, standard deviation 0.7 g). Animals were maintained under specific pathogen-free conditions.

HuH-7 (a well-differentiated hepatocellular carcinoma cell line) cells were obtained directly from the Japanese Collection of Research Bioresources Cell Bank (JCRB, Osaka, Japan). Cells were maintained in Dulbecco’s modified Eagle’s Medium (DMEM; Invitrogen, Carlsbad, CA, USA) with 10% fetal bovine serum (FBS; Invitrogen) at 37 °C in a humid atmosphere (5% CO_2_–95% air), and harvested by brief incubation in Enzyme-free Cell Dissociation Solution (Millipore Co., Bedford, MA, USA).

### ICG-NIR irradiation

For *in vitro* ICG-NIR irradiation, continuously cultured HuH-7 cells were harvested in tubes and resuspended in DMEM containing 10% FBS after washing with phosphate buffered saline (PBS; LSI Medience Co., Tokyo, Japan). The cells were seeded in triplicate in 96-well plates at a density of 2 × 10^4^ cells in 200 μL, and incubated at 37 °C in a 5% CO_2_ atmosphere. ICG (Diagnogreen; Daiichi Sankyo Co. Ltd., Tokyo, Japan) was administered 23 h after seeding. Forty-eight hours after seeding, ICG was removed from the culture medium and the cells were irradiated with 823-nm NIR laser light (prototype NIR Diode Laser System, Hamamatsu Photonics) at a power density of 160 mW/cm^2^ for 3 min. A second NIR irradiation was performed 24 h after the first irradiation.

For *in vivo* ICG-NIR irradiation, continuously cultured HuH-7 cells were harvested in tubes and resuspended in serum-free medium after washing with PBS. Each mouse was subcutaneously injected into the abdomen with 5 × 10^6^ HuH-7 cells in 200 μL of serum-free medium containing 50% Matrigel (Becton-Dickinson, Franklin Lakes NJ, USA). Ten days after transplantation, ICG (5 mg/kg) was administered to the mice intravenously via the tail vein. Twenty-four hours after injection (day 0), the mice were irradiated with 823-nm NIR laser light (prototype NIR Diode Laser System, Hamamatsu Photonics) (day 1) at three tumor locations. NIR irradiation was performed at a power density of 160 mW/cm^2^ for 3 min at each location. Total irradiation time was 9 min per mouse. After NIR irradiation, the mice were returned to the colony. Additional ICG was administered intravenously on day 6 and a second irradiation was performed on day 7. HuH-7 tumor sizes were measured every 3 days, and tumor volume was monitored for a total of 9 days following the first treatment. Tumor volume (mm^3^) was calculated using the ellipsoid volume equation: V = (π/6)*(d^2^*D), where d and D indicate the minor and major axes, respectively^19^.

### Temperature calibration study

The effect of laser irradiation on temperature in the tumor xenograft was analyzed. While the tumor xenograft was irradiated with 823-nm infrared laser light, temperature calibration studies were carried out using thermography to determine the temperature profile.

### TdT-mediated dUTP nick end labeling (TUNEL) analysis

TUNEL is a method for detecting DNA fragmentation by labeling the 3′-hydroxyl termini in the double-strand DNA breaks generated during apoptosis^20^. In this study, TUNEL analysis was performed to detect apoptosis in the HuH-7 tumor tissue. TUNEL staining was performed using an *in situ* cell death detection kit according to the manufacturer’s instruction (Roche Diagnostics GmbH, Penzberg, Germany).

### 3-(4,5-dimethylthiazol-2-yl)-2,5-diphenyltetrazolium bromide (MTT) assay

MTT is a monotetrazolium salt, which reflects cell proliferation and cytotoxicity^21^. The MTT assay involves the conversion of water soluble MTT to an insoluble formazan, which is then solubilized, and the absorbance of this solution is measured at 550 nm using a spectrophotometer^22^. In this study, cell viability was evaluated using an MTT assay. After 24-h incubation following NIR irradiation, thiazolyl blue tetrazolium bromide (Sigma-Aldrich, St. Louis, MO, USA) was added and incubated for an additional 4 h. Cells were then solubilized and absorbance at 550 nm was measured.

### Detection of oxidative stress

To evaluate the induction of oxidative stress *in vitro*, cytochemical analysis was performed using CellROX^®^ Green Reagent (Thermo Fisher Scientific, Waltham, MA). Thirty minutes to an hour after NIR irradiation, 5 μM CellROX^®^ Green Reagent was added and the cells were incubated for 30 min at 37 °C. Green fluorescent cells were detected by a fluorescence microscope (BZ-9000, Keyence, Osaka, Japan). Mean fluorescence intensity of cells was evaluated using Image J software (ImageJ 1.48 v; National Institutes of Health, Bethesda, MD, USA).

For *in vivo* analysis, ROS were evaluated through immunohistochemical analysis of 8-OHdG, a DNA marker of oxidative damage. Briefly, the formalin-fixed paraffin-embedded tissue sections of the HuH-7 tumor tissue were cut after NIR irradiation. After deparaffinization and re-hydration, sections were heated in a microwave for 5 min in 10 mM sodium citrate buffer (pH 6.0, LSI Medience Co., Tokyo, Japan) for antigen retrieval. The sections were then incubated with Anti-8-OHdG monoclonal antibody (10 μg/mL; Japan Institute for the Control of Aging, Nikken SEIL Corp, Shizuoka, Japan) overnight at 4 °C to detect the production of 8-OHdG. After the sections were incubated with a biotin-conjugated secondary antibody, a biotin-streptavidin-peroxidase complex method was performed using a commercial kit as per the manufacturer’s instructions (Vector Laboratories, Burlingame, CA). 3,3′-diaminobenzidine (Sigma-Aldrich, St. Louis, MO, USA) was used as the chromogen.

## Results

### Detection of heat production and apoptosis in the transplanted tumor tissue after ICG-NIR irradiation *in vivo*

The temperature in the transplanted tumor tissue after NIR irradiation was evaluated. The temperature of HuH-7 tumors increased to 48.5 °C in mice administered ICG and 43.4 °C in those that were not administered ICG.

**Figure 1:**
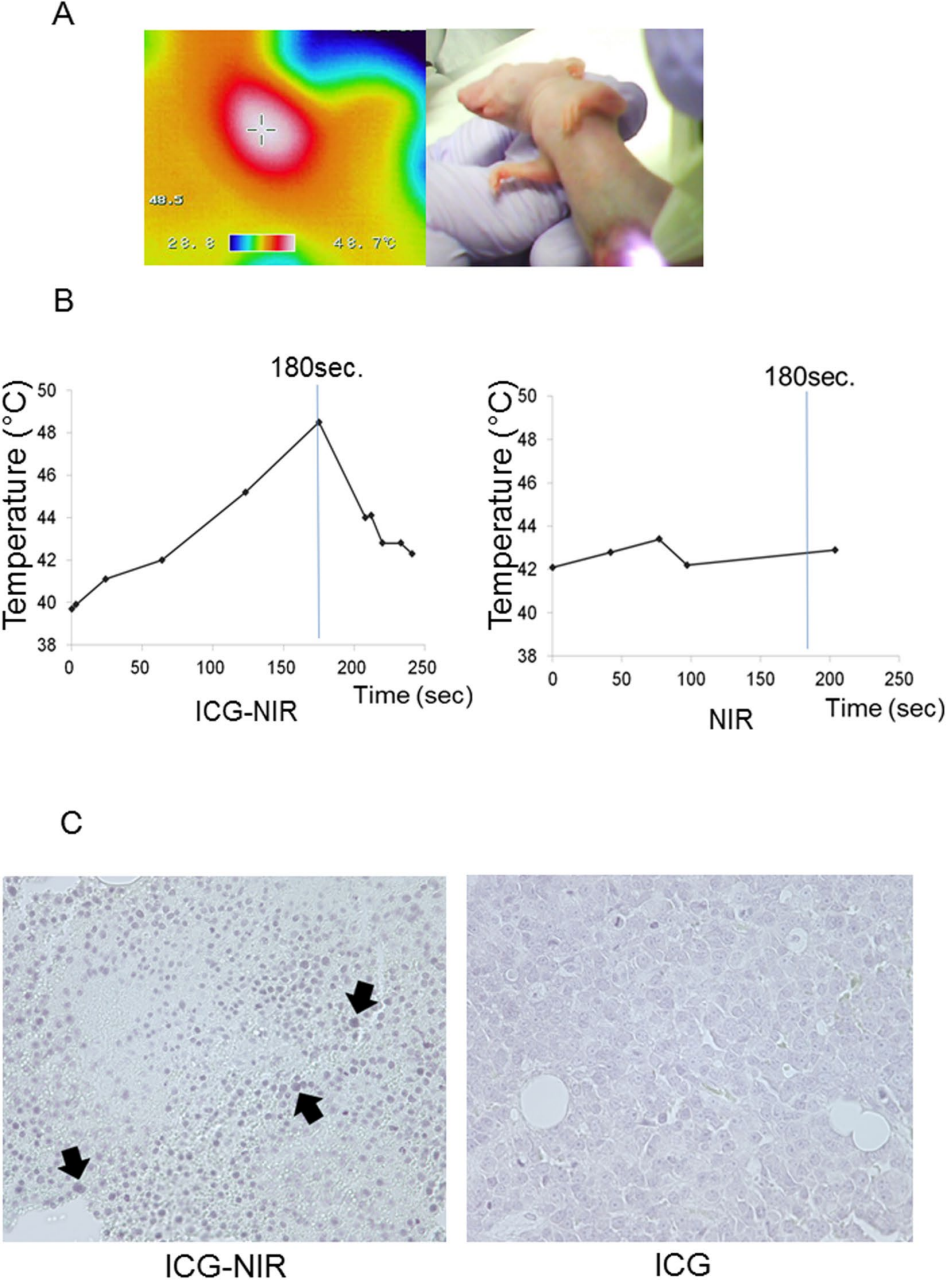
Detection of heat production and apoptosis in tumor xenograft model. (**A**) Temperature calibration analysis using thermography after ICG-NIR irradiation. (**B**) Time course of temperature elevation after NIR irradiation with or without ICG administration. (**C**) Detection of apoptosis through TUNEL staining (x 400). The intranuclear staining was evaluated. Arrows indicate the cells which express a typical apoptosis.

Furthermore, TUNEL analysis detected apoptosis in tumor tissue after ICG-NIR irradiation, while apoptosis was not detected in only ICG group. (Fig. 1C). These results suggested that the photothermal effects contribute to the anti-tumor effect of ICG-NIR.

### Anti-tumor effect of ICG-NIR under cooling conditions *in vitro*

**Figure 2:**
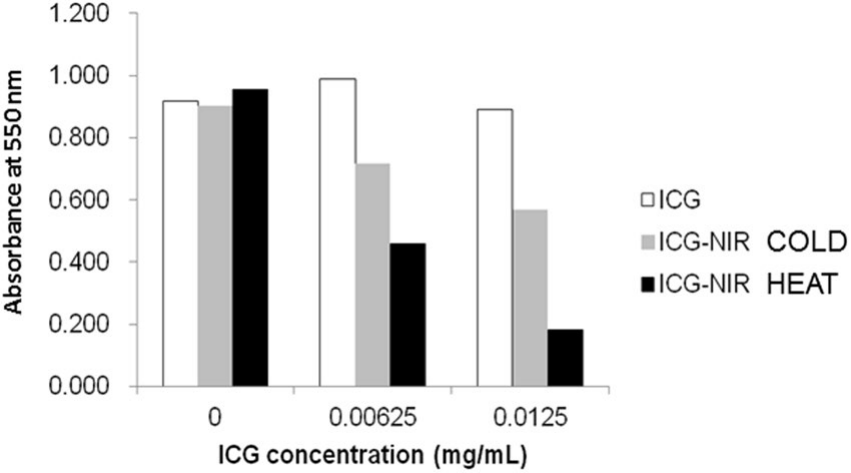
MTT assay with or without heat storage sheet after ICG-NIR *in vitro*. HEAT: ICG-NIR irradiation on the heat storage sheet, COLD: ICG-NIR irradiation on the cooling sheet. Higher absorbance at 550 nm reflects higher cell viability.

To clarify the role of photothermal effects in the anti-tumor effect of ICG-NIR, we examined cell viability after ICG-NIR on a heat storage sheet and on a cooling sheet, which inhibits the temperature elevation. As shown in Fig. 2, the anti-tumor effect of ICG-NIR was inhibited by the cooling sheet, indicating that heat is an essential mechanism for the anti-tumor effect of ICG-NIR. On the other hand, a partial anti-tumor effect of ICG-NIR was observed on the cooling sheet. We have hypothesized this effect was due to ROS production by ICG-PDT.

### Production of ROS after ICG-NIR irradiation

**Figure 3:**
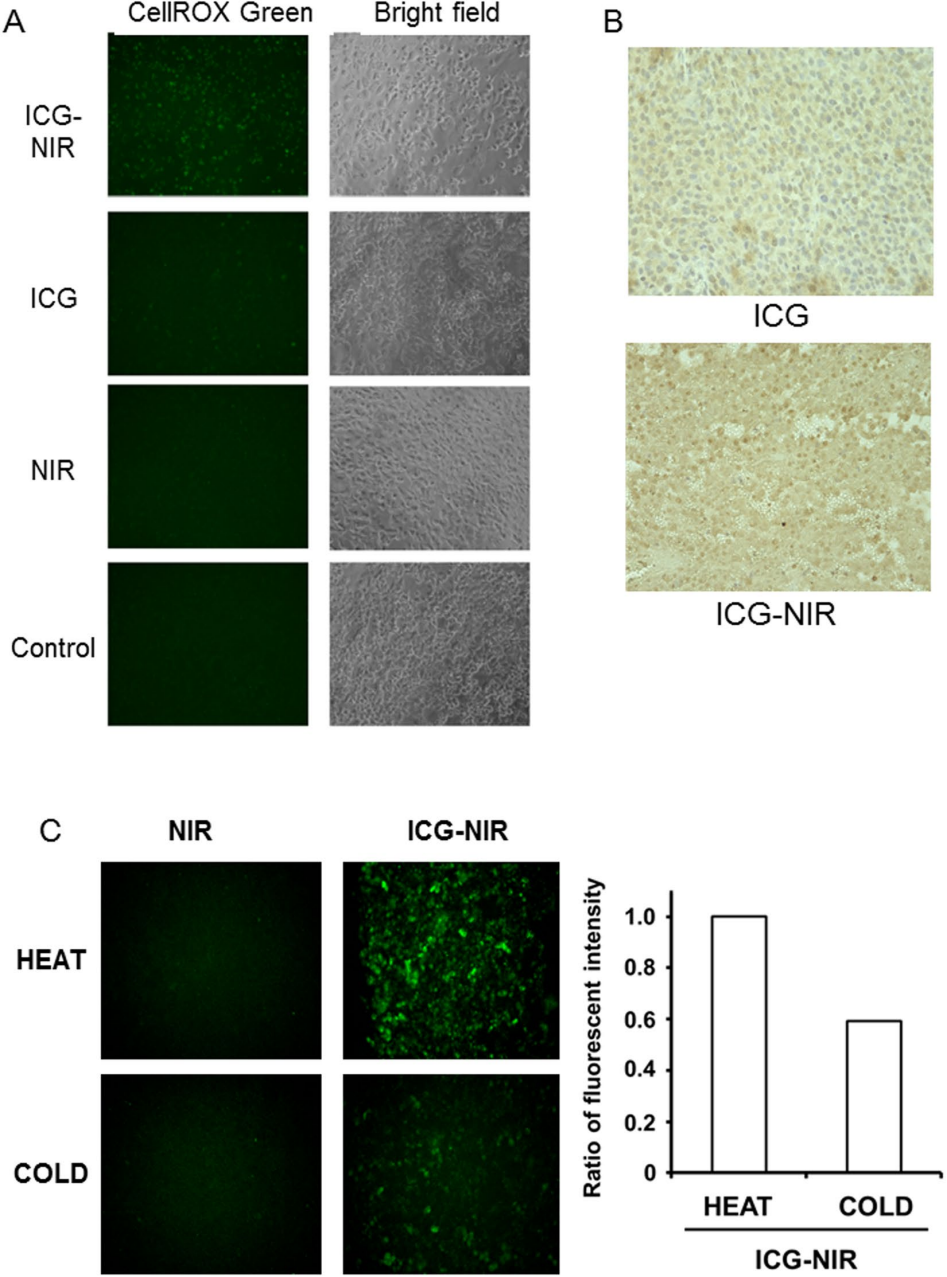
Detection of ROS production. (**A**) Detection of CellRox Green fluorescence after ICG-NIR *in vitro* (x200). (**B**) Immunohistochemical analysis of 8-OHdG after ICG-NIR in tumor xenograft model (x400). 8-OHdG is a DNA marker of oxidative damage and positive staining of 8-OHdG indicates ROS production. (**C**) Detection of CellRox Green fluorescence and the ratio of fluorescence intensity of ICG-NIR with or without heat storage sheet *in vitro* (x200). HEAT: with heat storage sheet, COLD: without heat storage sheet.

ROS production was strongly detected in ICG-NIR-irradiated cells compared to cells treated with ICG alone or NIR alone, or control cells *in vitro* (Fig. 3A). To analyze the production of ROS by ICG-NIR irradiation *in vivo*, we evaluated the expression of 8-OHdG in the xenograft tumor. Positive staining of 8-OHdG was detected in tumor sections after ICG-NIR irradiation, but not in tumor sections after ICG administration alone (Fig. 3B). Furthermore, ROS production of ICG-NIR was inhibited under the cooling condition *in vitro* (Fig. 3C).

### Suppression of HCC cell growth by repeated NIR irradiation

**Figure 4:**
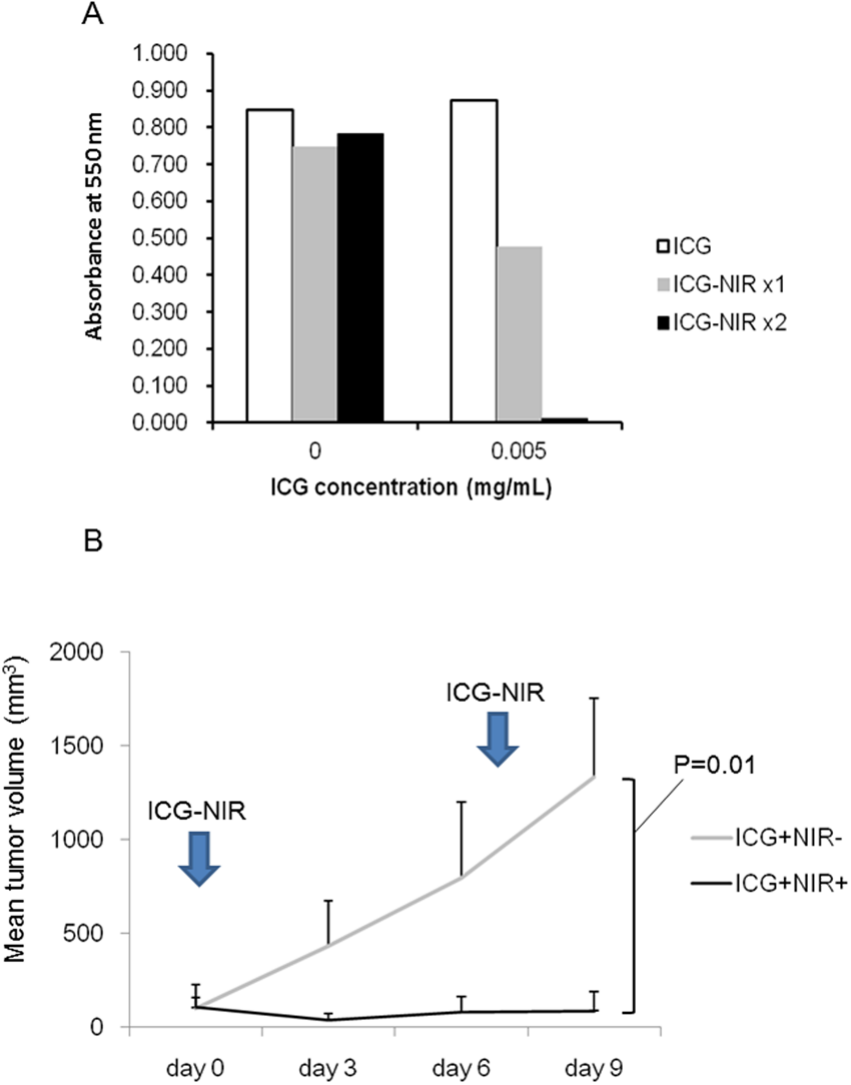
Repeated ICG-NIR irradiation. (**A**) MTT assay after repeated ICG-NIR *in vitro*. Higher absorbance at 550 nm reflects higher cell viability. (**B**) Tumor volume growth after repeated ICG-NIR in tumor xenograft model.

Photothermal effects and oxidative stress were found to be the essential mechanisms underlying ICG-NIR therapy, and thus, we hypothesized that repeated NIR irradiation may improve the anti-tumor effect of ICG-NIR. Indeed, the anti-tumor effect was significantly enhanced after repeated NIR irradiation *in vitro* (Fig. 4A). We further evaluated the effect of repeated ICG-NIR irradiation in an HCC mouse xenograft model. The mean tumor volume of the ICG-administered control mice (n = 5) steadily increased from 99 mm^3^ on day 0 to 1332 mm^3^ on day 9. In contrast, the mean tumor volume growth was completely suppressed in the ICG-NIR mice (n = 5) from 104 mm^3^ on day 0 to 87 mm^3^ by day 9 (P = 0.01) (Fig. 4B).

## Discussion

**Figure 5:**
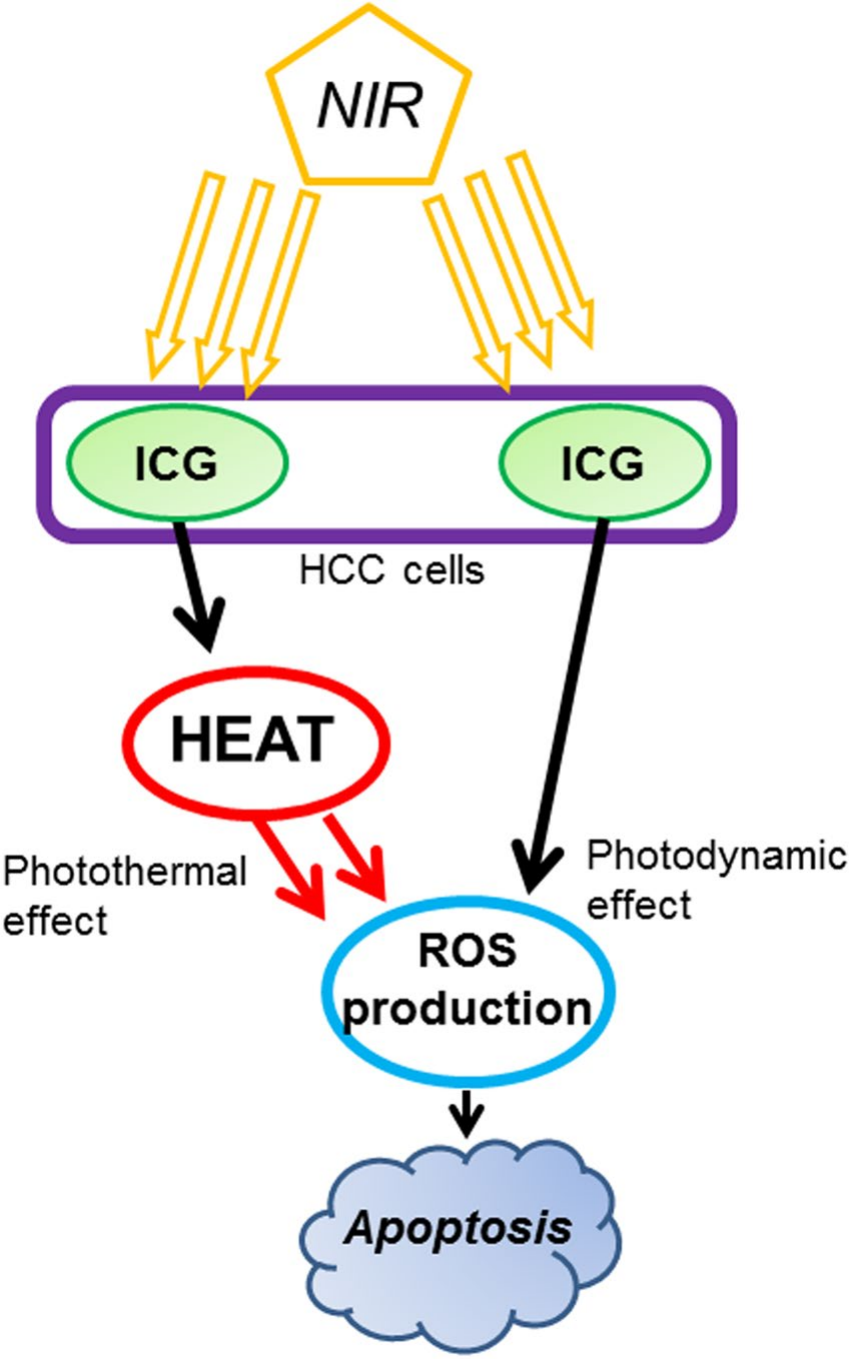
Anti-tumor mechanism of ICG-NIR.

The results of the present study indicated that the apoptosis of HCC cells after ICG-NIR therapy is mediated by photothermal effect and oxidative stress induced by PDT, both *in vitro* and *in vivo* (Fig. 5). Moreover, HuH-7 cell toxicity induced by ICG-NIR therapy depends on the frequency of exposure to NIR laser.

ICG is an effective absorber of laser-mediated PTT^23^. Reindl *et al*.^24^ reported that ICG absorbs 800-nm NIR light, and approximately 88% of the absorbed light is converted to heat. In our study, ICG-NIR produced heat up to 48.5 °C during NIR light irradiation *in vivo*. The anti-tumor effect was attenuated under heat suppression *in vitro*. These results suggested that the photothermal effect plays an important role in the anti-tumor effect of ICG-NIR.

Because photothermal effect and oxidative stress were the key mechanisms underlying the anti-tumor effect of ICG-NIR therapy, we examined the effect of repeated ICG-NIR. Repeated NIR laser exposure almost completely abolished the viability of HuH-7 cells *in vitro*. Furthermore, the growth of explanted tumor cells was completely inhibited after repeated NIR light irradiation *in vivo*, which was not observed after single ICG-NIR irradiation^18^. These findings indicated that the effect of ICG-NIR therapy on HCC depends on the NIR laser exposure frequency. We previously reported^18^ that NIR irradiation alone, under the same condition as that in the present report, does not suppress tumor growth, which indicates that ICG-NIR therapy needs ICG as photothermal agent and photosensitizer.

PDT generates singlet oxygen and the energy absorption of ICG from NIR creates an activated form of singlet oxygen^25,26^. This singlet oxygen reacts rapidly with cellular components to cause the damage that ultimately leads to cell death^27^. In our study, ROS production was also detected after ICG-NIR irradiation both *in vitro* and *in vivo*. Although ICG is approved by the USFDA only as a contrast agent, it has been repeatedly reported to act as a photosensitizer^8,18,25–27^. In our study, ICG-NIR group exhibited some anti-tumor effect even under cooling conditions, which indicates that ICG-PDT also contributes to the anti-tumor effect of ICG-NIR.

The major advantage of ICG-NIR therapy is its high selectivity for HCC tissues. Usually, photosensitizers have relatively weak tumor selectivity. For example, the fluorescence intensity of 5-aminolaevulinia is only 1.4 times higher than that in the background in non-tumor tissues^28^. Mitsunaga *et al*. reported that the tumor-to-background ratio of the photosensitizer IRDye 700DX N-hydroxysuccinimide ester is 7:1^29^. We previously reported that ICG fluorescence has an extremely high tumor-to-background ratio (255:1) using a fluorescence imaging system^18^. Therefore, the effect of ICG-NIR therapy on the surrounding healthy tissue will be minimal.

After intravenous injection, the main peak of the absorption spectrum of ICG shifts ~25 nm toward a higher wavelength, from 780 to 805 nm. This shift corresponds to the change in the physicochemical environment of the molecules within seconds after the injection^9,30,31^. In the present study, we used a Diode Laser System with 823-nm wavelength infrared laser light, which do not quite reach the maximum absorption wavelength of ICG in the living body. The effect of ICG-NIR therapy might therefore be improved by using NIR close to 805 nm.

Curative treatments for HCC include surgical resection, liver transplantation, and ablation therapy^14,32^. However, these treatments are often not applicable in patients with multiple lesions due to many reasons, including the patient’s liver function and technical difficulties^14,32^. The ICG-NIR therapy can be applied to both tumor detection and anti-tumor therapy. Thus, surgeons can identify residual tumors using the ICG fluorescence technique, and ICG-NIR therapy can be simultaneously performed as an additional therapy to the residual tumor tissue. ICG-NIR therapy may also be applied to disseminated peritoneal lesions of HCC. Other advantages of ICG-NIR therapy include its safety and feasibility. Due to the high selectivity of ICG for HCC tumors, ICG-NIR therapy causes minimal damage to surrounding healthy liver tissues. The major limitation of the present findings for clinical application is the limited penetration depth of NIR light. The reported penetration depth of NIR light is approximately 10 mm, and ICG-NIR therapy is thought to be an effective therapeutic tool only for tumors on the liver surface or for peritoneal dissemination^12,33–35^. A laparotomy or laparoscopic surgery may be necessary for the clinical use of ICG-NIR therapy in HCC treatment.

In conclusion, the anti-tumor effect of ICG-NIR therapy against human HCC is due to apoptosis through photothermal effect and oxidative stress. These effects could be enhanced by repeated ICG-NIR irradiation.
